# Supplementary material for: mRNA mediates passive vaccination against infectious agents, toxins, and tumors
Source: EMBO Mol Med. 2017 Aug 9;9(10):1434–47. doi: 10.15252/emmm.201707678 (PMC5623855; doi:10.15252/emmm.201707678)
Supplement: Supplementary file 1 — Appendix [file EMMM-9-1434-s001.pdf]

Appendix

mRNA mediates passive vaccination against infectious agents, toxins and tumors

Moritz Thran, Jean Mukherjee, Marion Pönisch, Katja Fiedler, Andreas Thess, Barbara L. Mui, Michael J. Hope, Ying K. Tam, Nigel Horscroft, Regina Heidenreich, Mariola Fotin-Mleczek, Charles B. Shoemaker, Thomas Schlake

This appendix includes 10 figures and 4 tables

		Page
**Table of Contents**		1
**Appendix Figure S1.**	Solitary heavy chain molecules are retained in the cytoplasm.	2
**Appendix Figure S2.**	Titration of the molar ratio of heavy and light chain-encoding mRNAs.	3
**Appendix Figure S3.**	Various mAbs can be expressed by exogenous mRNA *in vitro*.	4
**Appendix Figure S4.**	Kinetics of mRNA-LNP expression *in vitro* and *in vivo*.	*5*
**Appendix Figure S5.**	Cartoon illustration of the assay for measuring antigen-binding by mRNA-encoded mAbs.	6
**Appendix Figure S6.**	Inhibition of HIV cell entry determined by a Magi R5-Tropic Antiviral Assay.	7
**Appendix Figure S7.**	*In vivo* expression analyses of anti-influenza B mAb and VNAs.	8
**Appendix Figure S8.**	Correlation of IgG titers and ADA responses.	9
**Appendix Figure S9.**	Histopathological analysis of liver sections after mRNA-LNP treatment.	10
**Appendix Figure S10.**	Protection of mice against BoNT/A challenge by mRNA-LNP administered at various times post intoxication.	11
**Appendix Table S1.**	Analysis of cytokine induction following mRNA-LNP administration.	12
**Appendix Table S2.**	Supporting information Fig. 4b.	13
**Appendix Table S3.**	Supporting information Fig. 4d.	14
**Appendix Table S4.**	Supporting information Fig. 4e.	15-16

Appendix Figure S1

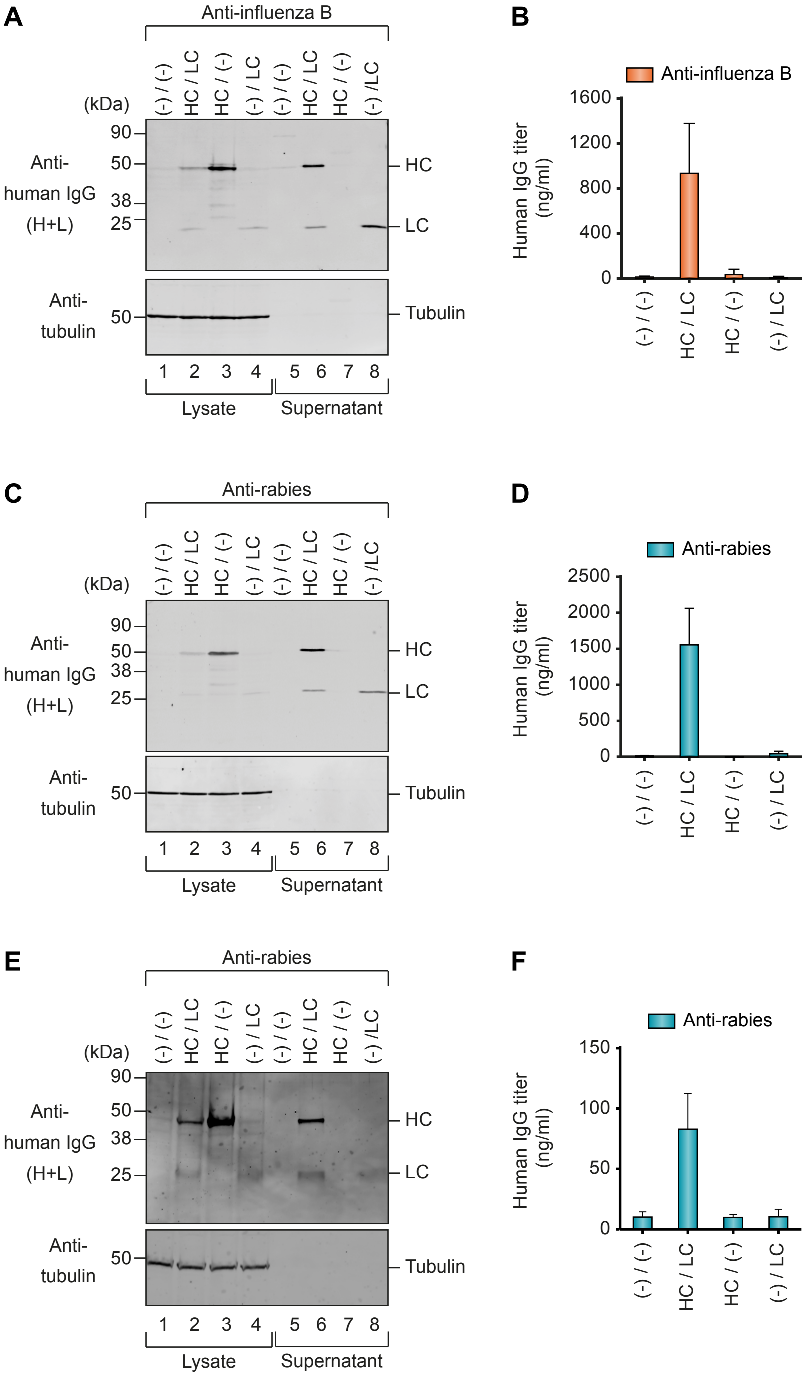

Appendix Figure S1. Solitary heavy chain molecules are retained in the cytoplasm.

A, C, E mRNAs encoding heavy chain (HC) and light chain (LC), respectively, were transfected in different combinations into BHK (A and C) or HepG2 (E) cells and expression profiles were analyzed from lysates and supernatants by western blot. Equal amounts of three replicates were pooled and loaded on denaturing SDS-PAGE followed by western blot analysis.

B, D, F Individual supernatants (triplicates) that were pooled for (A), (C) and (E) were analyzed by IgG-specific ELISA. For mock co-transfections (indicated as (-)), an mRNA encoding eGFP was used.

Data information: Data in B, D and F are expressed as means ± SD.

Appendix Figure S2

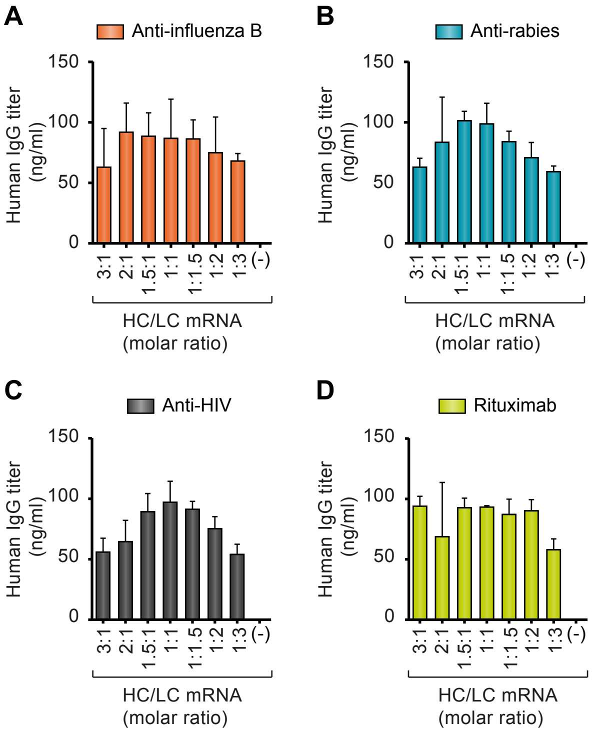

Appendix Figure S2. Titration of the molar ratio of heavy and light chain-encoding mRNAs.

A, B, C, D — Quantification of mAb levels from BHK cell supernatants by IgG-specific ELISA. BHK cells were transfected with various ratios of heavy and light chain-encoding mRNAs giving rise to mAbs against influenza B (A), rabies (B), HIV (C), or human CD20 (D). Measurements were conducted in quadruplicate.

Data information: Data are expressed as means ± SD.

Appendix Figure S3

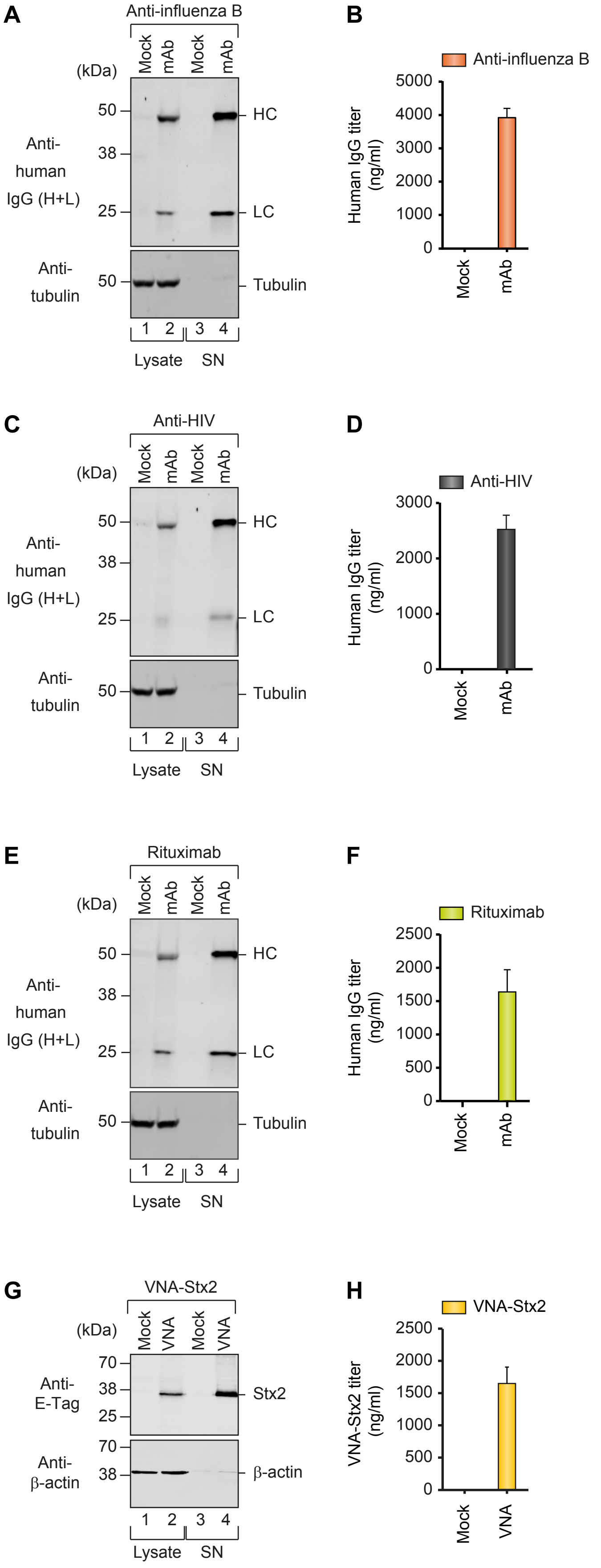

Appendix Figure S3. Various antibodies can be expressed by exogenous mRNA in vitro.

A, C, E, G	mRNAs encoding distinct antibodies were transfected into BHK cells and expression was analyzed in lysates and supernatants by denaturing SDS-PAGE followed by western blot analysis. Equal amounts of three replicates were pooled for gel electrophoresis.
B, D, F, H	Quantification of titers from supernatants of BHK cells by IgG- (B, D, F) or antigen-specific ELISA (H). Titers were determined in biological triplicates.

Data information: Data in B, D, F and H are expressed as means ± SD.

Appendix Figure S4

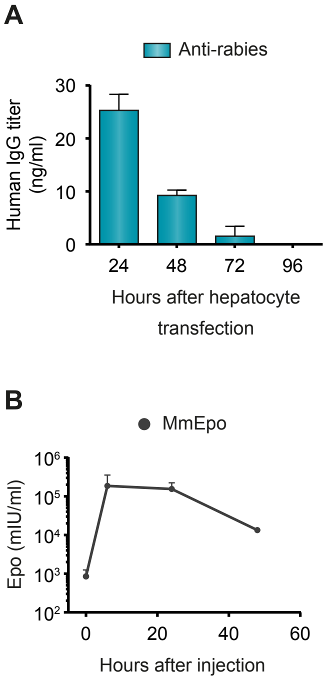

Appendix Figure S4. Kinetics of mRNA-LNP expression in vitro and in vivo.

A Primary mouse hepatocytes were transfected with mRNA-LNP encoding anti-rabies mAb. Expression of mAb at various times was determined in biological duplicates by IgG-specific ELISA.

B Mice received 30 µg of mRNA-LNP encoding MfEpo. Expression of MfEpo at various times was determined in biological duplicates by ELISA.

Data information: Data are expressed as means ± SD.

Appendix Figure S5

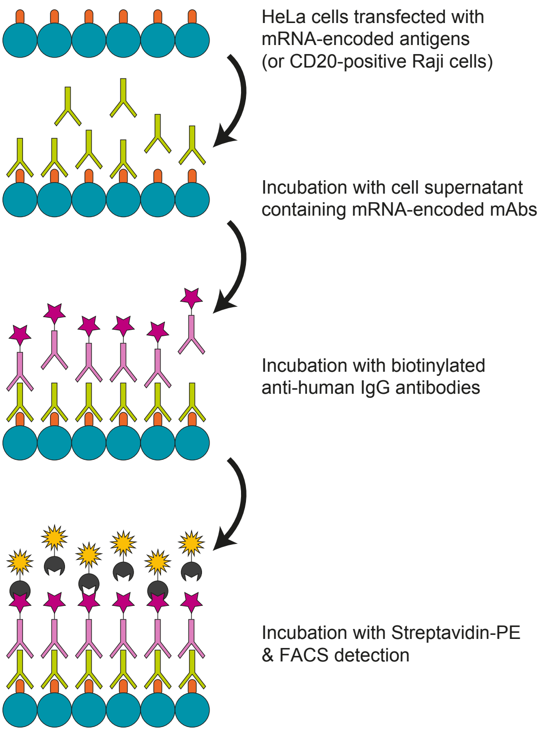

Appendix Figure S5. Cartoon illustration of the assay for measuring antigen-binding by mRNA-encoded mAbs.

If no natural target cells were available, HeLa cells were transfected to express the cognate antigen of the mAb of interest. Following incubation with mAb-containing supernatants from BHK cells, Hela or Raji cells were further incubated with anti-human IgG specific biotinylated antibody and finally incubated with streptavidin-coupled phycoerythrin (PE).
Binding specificity correlates with PE intensity and can be assessed by semi-quantitative FACS analysis.

Appendix Figure S6

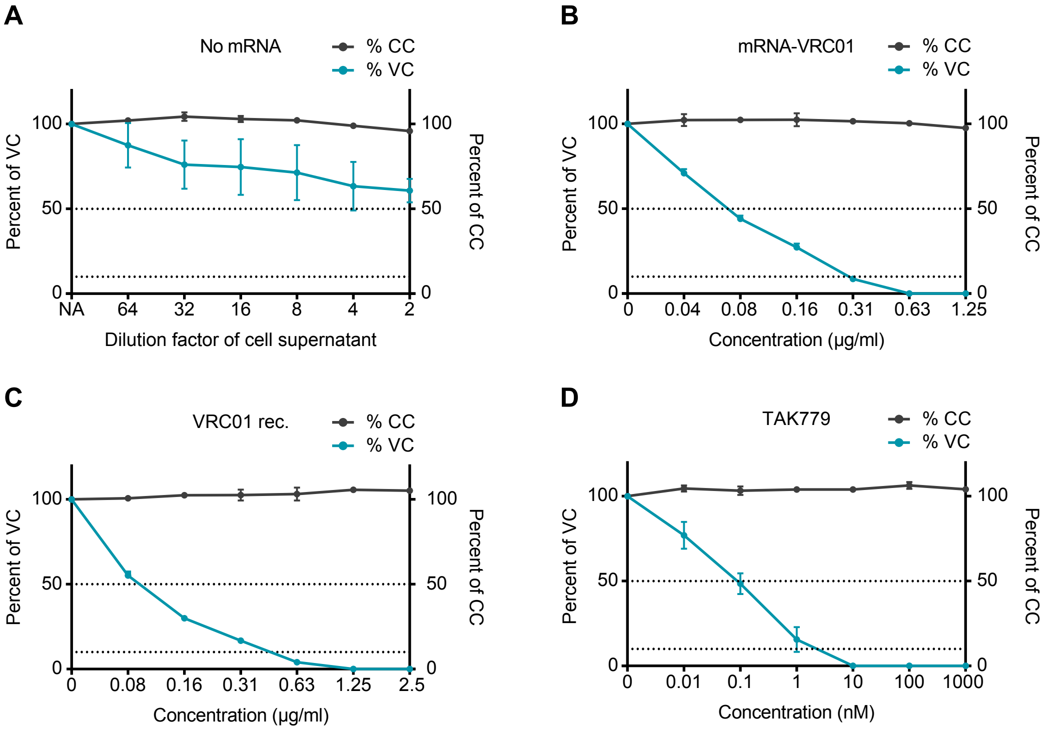

Appendix Figure S6. Inhibition of HIV cell entry determined by a Magi R5-Tropic Antiviral Assay.

A, B, C, D — mRNA encoding VRC01 was transfected into BHK cells in triplicates. BHK supernatants were pooled and added to MAGI-R5 cells before Ba-L strain of HIV-1 was then added to the cells. Virus entry was measured via chemiluminescence. Depicted is the ratio of chemiluminescence compared to a virus control (VC) which represents infected cells in the absence of any test compound. Cell control (CC) measurements were carried out to analyze cell viability at different dilutions of medium in the absence of virus. A serial dilution of supernatants of untransfected cells (A), cells transfected with RNA encoding VRC01 (B), recombinant VRC01 (C) and the TAK779 inhibitor (D) was carried out and the virus entry was measured. Dotted lines indicate an inhibition of 50% (IC_{50}) and of 90% (IC_{90}) compared to virus control.

Data information: Data are expressed as means ± SD.

Appendix Figure S7

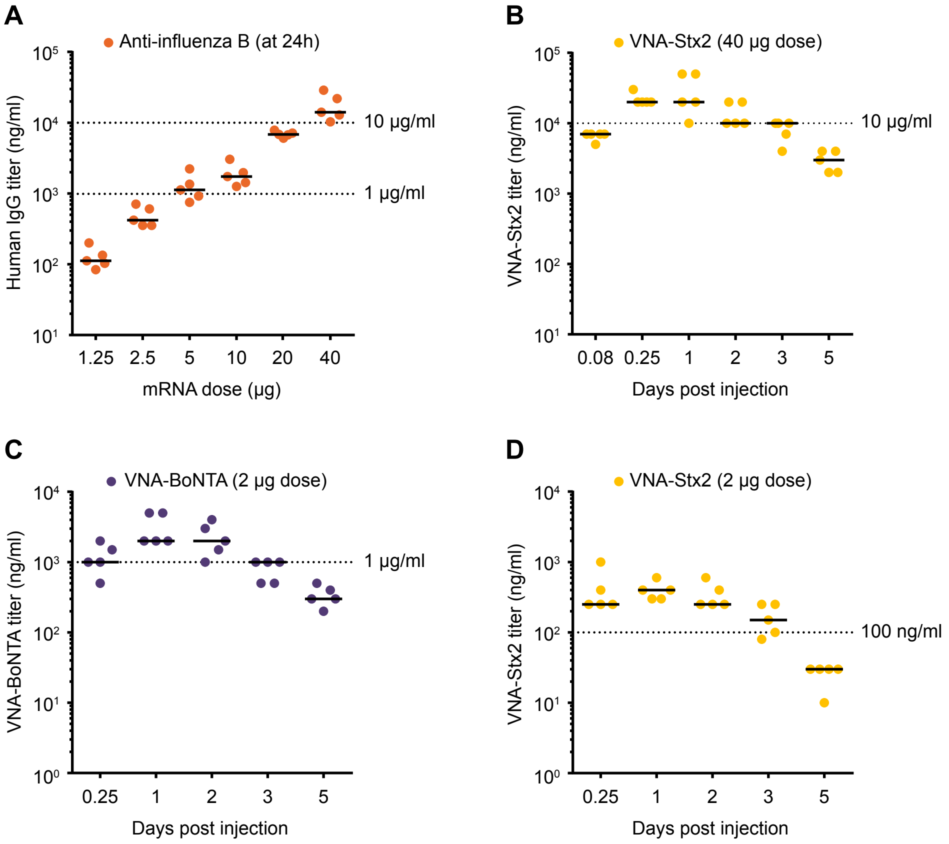

Appendix Figure S7. In vivo expression analyses of anti-influenza B mAb and VNAs.

A Quantification of mAb titers by IgG-specific ELISA of sera obtained 24 hours after a single intravenous injection of increasing doses of mRNA-LNP encoding anti-influenza B mAb into mice.

B Quantification of VNA titers by antigen-specific ELISA at various times after a single intravenous injection of 40 µg of mRNA-LNP encoding VNA-Stx2. For each time point, a different cohort of mice was used.

C, D Quantification of VNA titers by antigen-specific ELISA at various times, following a single intravenous injection of 2 µg of mRNA-LNP encoding either VNA-BoNTA or VNA-Stx2.
For each time point a different cohort of mice was used.

Data information: Individual measurements as well as means are given for each time point.

Appendix Figure S8

A

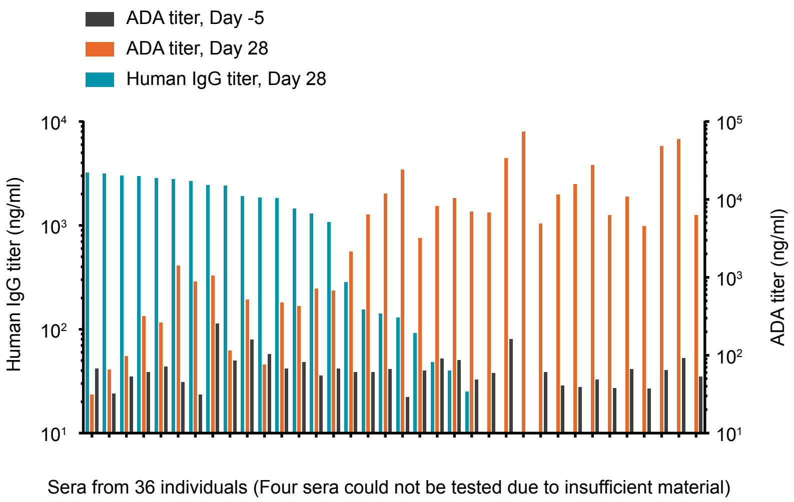

Correlation:	Human IgG titer (Day 28) / ADA titer (Day 28)
Spearman r:	- 0.8415
P (two tailed):	< 0.0001

Appendix Figure S8. Correlation of IgG titers and ADA responses.

All animals used for analysis of antibody expression kinetics (Fig. 3C) were bled on day 28 and antibody titers were determined using an IgG specific ELISA. ADA titers were determined by an ADA ELISA that specifically detects humoral responses against the anti-rabies antibody using day -5 (pre-bleed) and day 28 samples. Correlation of antibody and ADA titers was determined by two-tailed spearman correlation analysis.

Data information: Bars represent individual measurements.

Appendix Figure S9

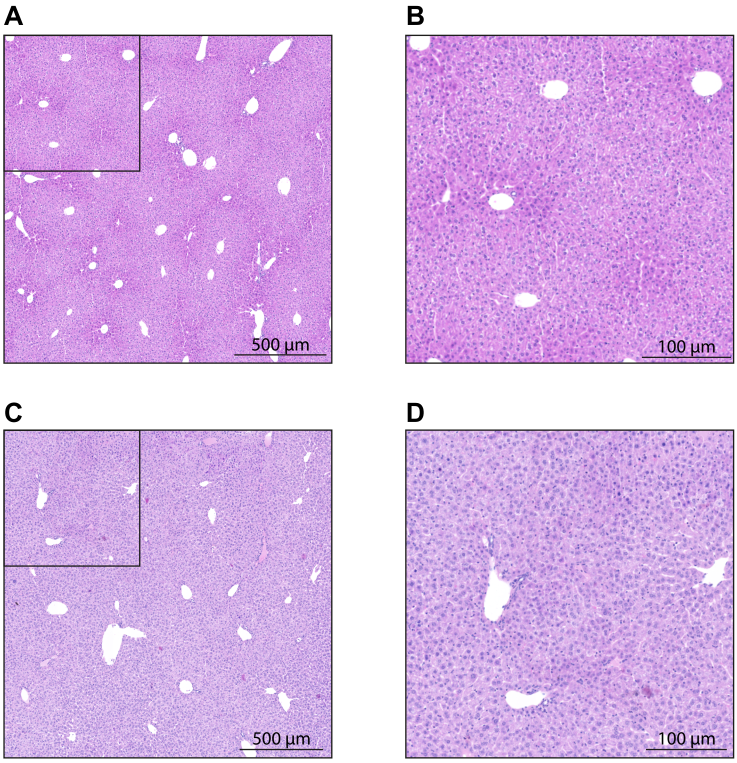

Appendix Figure S9. Histopathological analysis of liver sections after mRNA-LNP treatment.

A, B Histopathological analysis of liver sections of untreated mice at high (A) and low (B) magnification.

C, D Histopathological analysis of liver sections of mice after mRNA-LNP treatment at high (C) and low (D) magnification.

Appendix Figure S10

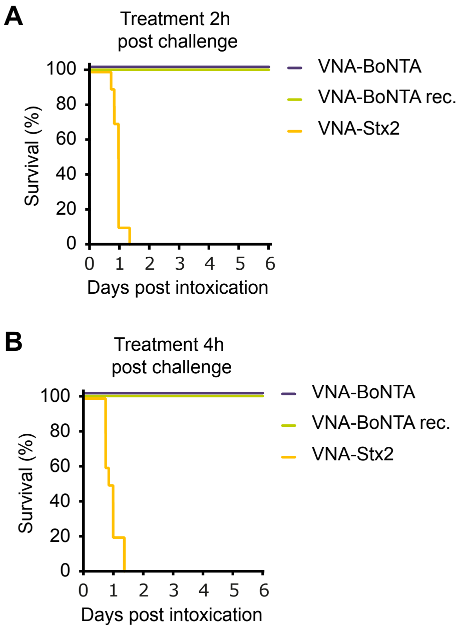

Appendix Figure S10. Protection of mice against BoNT/A challenge by mRNA-LNP administered at various times post intoxication.

A, B CD1 mice received a single intravenous injection of 2 µg of recombinant VNA-BoNTA or 40 µg of mRNA-LNP encoding either VNA-BoNTA or VNA-Stx2 at two (A) or four (B) hours after a lethal 4x LD50 intravenous BoNT/A challenge. Survival was monitored for six days. A total of ten mice per group were challenged.

Appendix Table S1

Analysis of cytokine induction following mRNA-LNP administration

mRNA-LNP dose	40 µg (2 mg/kg)			
Cytokine (pg/ml)	TNF	IL-6	GM-CSF	IL-12p70
Day -5	2,8 ± 1	1,9 ± 1	3,1 ± 6,1	Not detected
6 hours	80,9 ± 19,8	298,8 ± 92,1	8,9 ± 6,1	Not detected

Appendix Table S2

Supporting information Fig. 4b

Scores	
0	no clinical signs
1	early sign of rabies encephalitis: ruffled fur, excessive grooming, restless / overly active, coordination problems (unsteady gait), blinking/squeezing of eyes, arched back
2	progression to rabies encephalitis: ruffled fur, impaired use of one of the hind legs, inflexible tail, hunchback
3	rabies encephalitis **(humane endpoint)**: impaired use of the hind quarter, rolls over when walking, inability to stand up, hunchback, inflexible tail / curled upwards

Body Weight (BW)	
< 90 % of Day 0	
< 85 % of Day 0	
< 80 % of Day 0	Humane endpoint was also reached if the weight of mice reduced more than 5 % on two following days.

			Day after Challenge																					
	**Mouse**	**Parameter**	**0**	**1**	**2**	**3**	**4**	**5**	**6**	**7**	**8**	**9**	**10**	**11**	**12**	**13**	**14**	**15**	**16**	**17**	**18**	**19**	**20**	**21**
**Anti-rabies (40ug) Day -1**	**#1**	**Score**	0	0	0	0	0	0	0	0	0	0	0	0	0	0	0	0	0	0	0	0	0	0
		**BW**	100	100	108	108	108	106	105	107	112	114	112	111	113	114	116	118	120	119	121	122	120	119
	**#2**	**Score**	0	0	0	0	0	0	0	0	0	0	0	0	0	0	0	0	0	0	0	0	0	0
		**BW**	100	101	109	111	108	108	109	107	112	111	111	114	114	114	114	116	117	119	118	117	123	121
	**#3**	**Score**	0	0	0	0	0	0	1	0	0	0	0	0	0	0	0	0	0	0	0	0	0	0
		**BW**	100	101	108	108	109	107	108	110	115	114	113	116	115	115	116	118	117	116	119	120	116	113
	**#4**	**Score**	0	0	0	0	0	0	0	0	0	0	0	0	0	0	0	0	0	0	0	0	0	0
		**BW**	100	102	109	107	106	107	107	104	106	108	108	109	109	109	110	112	110	106	106	110	110	109
	**#5**	**Score**	0	0	0	1	0	1	1	1	1	1	2	1	1	1	1	1	1	1	1	1	1	1
		**BW**	100	101	110	104	101	101	98	97	104	104	101	104	104	108	107	107	108	107	107	109	113	113
	**#6**	**Score**	0	0	0	0	0	0	0	0	0	0	0	0	0	0	0	0	0	0	0	0	0	0
		**BW**	100	102	112	112	108	107	109	108	112	110	109	112	114	113	114	115	119	118	119	115	116	114
**Anti-influenza B (40ug) Day -1**	**#1**	**Score**	0	0	0	0	0	0	0	0	1	**3**	N/A	N/A	N/A	N/A	N/A	N/A	N/A	N/A	N/A	N/A	N/A	N/A
		**BW**	100	99	105	109	109	107	108	100	94	**87**												
	**#2**	**Score**	0	0	0	0	0	0	1	**3**	N/A	N/A	N/A	N/A	N/A	N/A	N/A	N/A	N/A	N/A	N/A	N/A	N/A	N/A
		**BW**	100	100	106	108	109	108	99	**91**														
	**#3**	**Score**	0	0	0	0	0	1	2	**3**	N/A	N/A	N/A	N/A	N/A	N/A	N/A	N/A	N/A	N/A	N/A	N/A	N/A	N/A
		**BW**	100	101	107	112	109	98	90	**81**														
	**#4**	**Score**	0	0	0	0	0	0	0	0	0	0	1	0	0	0	1	2	**2**	N/A	N/A	N/A	N/A	N/A
		**BW**	100	102	104	110	111	111	109	110	109	111	98	92	89	85	79	73	**67**					
	**#5**	**Score**	0	0	0	0	0	1	2	**3**	N/A	N/A	N/A	N/A	N/A	N/A	N/A	N/A	N/A	N/A	N/A	N/A	N/A	N/A
		**BW**	100	98	106	107	106	95	86	**79**														
	**#6**	**Score**	0	0	0	0	0	1	1	**3**	N/A	N/A	N/A	N/A	N/A	N/A	N/A	N/A	N/A	N/A	N/A	N/A	N/A	N/A
		**BW**	100	100	104	107	108	107	91	**85**														

Appendix Table S3

Supporting information Fig. 4d

Scores	
0	no clinical signs
1	early sign of rabies encephalitis: ruffled fur, excessive grooming, restless / overly active, coordination problems (unsteady gait), blinking/squeezing of eyes, arched back
2	progression to rabies encephalitis: ruffled fur, impaired use of one of the hind legs, inflexible tail, hunchback
3	rabies encephalitis **(humane endpoint)**: impaired use of the hind quarter, rolls over when walking, inability to stand up, hunchback, inflexible tail / curled upwards

Body Weight (BW)	
< 90 % of Day 0	
< 85 % of Day 0	
< 80 % of Day 0	Humane endpoint was also reached if the weight of mice reduced more than 5 % on two following days.

			Day after Challenge																					
	**Mouse**	**Parameter**	**0**	**1**	**2**	**3**	**4**	**5**	**6**	**7**	**8**	**9**	**10**	**11**	**12**	**13**	**14**	**15**	**16**	**17**	**18**	**19**	**20**	**21**
**Anti-rabies (40ug) +2 hours**	**#1**	**Score**	0	0	0	0	0	0	0	0	0	0	0	0	0	0	0	0	0	0	0	0	0	0
		**BW**	100	93	93	98	101	102	104	101	107	106	107	109	109	110	111	112	112	115	115	115	120	117
	**#2**	**Score**	0	0	0	0	0	0	0	1	1	1	0	0	0	0	1	1	1	1	0	0	1	1
		**BW**	100	95	96	103	100	100	97	91	92	100	101	106	102	108	106	104	102	103	102	103	104	104
	**#3**	**Score**	0	0	0	0	0	0	0	1	0	0	0	0	0	0	0	0	0	0	0	0	0	0
		**BW**	100	94	94	99	99	99	100	100	105	105	105	106	104	107	104	107	108	111	109	110	111	111
	**#4**	**Score**	0	0	0	0	0	0	0	0	0	0	0	0	0	0	0	0	0	0	0	0	0	0
		**BW**	100	96	100	104	106	104	106	101	103	103	101	102	101	104	102	102	101	103	103	104	105	106
	**#5**	**Score**	0	0	0	0	0	0	0	0	0	0	0	0	0	0	0	0	0	0	0	0	0	0
		**BW**	100	95	96	99	101	98	98	100	102	102	103	103	103	103	102	105	104	105	108	108	111	111
	**#6**	**Score**	0	0	0	0	0	0	0	1	0	0	0	0	0	0	0	0	0	0	0	0	0	0
		**BW**	100	94	96	100	97	96	97	96	101	99	99	102	100	102	100	102	101	103	107	105	107	105
**Anti-influenza B (40ug) +2 hours**	**#1**	**Score**	0	0	0	0	0	2	**3**	N/A	N/A	N/A	N/A	N/A	N/A	N/A	N/A	N/A	N/A	N/A	N/A	N/A	N/A	N/A
		**BW**	100	97	98	103	103	94	**87**															
	**#2**	**Score**	0	0	0	0	0	1	2	2	**2**	N/A	N/A	N/A	N/A	N/A	N/A	N/A	N/A	N/A	N/A	N/A	N/A	N/A
		**BW**	100	93	96	100	96	93	84	76	**71**													
	**#3**	**Score**	0	0	0	0	0	0	0	1	0	0	1	0	1	1	1	1	1	1	1	1	1	1
		**BW**	100	90	91	97	100	100	101	101	105	100	90	86	85	86	85	84	85	84	85	93	91	93
	**#4**	**Score**	0	0	0	0	0	1	**3**	N/A	N/A	N/A	N/A	N/A	N/A	N/A	N/A	N/A	N/A	N/A	N/A	N/A	N/A	N/A
		**BW**	100	94	94	100	100	88	**82**															
	**#5**	**Score**	0	0	0	0	0	0	1	0	0	0	0	0	0	0	0	0	0	0	0	0	0	0
		**BW**	100	96	95	100	101	100	99	98	101	101	99	99	98	97	100	100	102	101	103	104	102	102
	**#6**	**Score**	0	0	0	0	0	1	2	**3**	N/A	N/A	N/A	N/A	N/A	N/A	N/A	N/A	N/A	N/A	N/A	N/A	N/A	N/A
		**BW**	100	95	97	100	101	95	89	**83**														

Appendix Table S4

Supporting information Fig. 4e

Scores	
0	no clinical signs
1	early sign of rabies encephalitis: ruffled fur, excessive grooming, restless / overly active, coordination problems (unsteady gait), blinking/squeezing of eyes, arched back
2	progression to rabies encephalitis: ruffled fur, impaired use of one of the hind legs, inflexible tail, hunchback
3	rabies encephalitis **(humane endpoint)**: impaired use of the hind quarter, rolls over when walking, inability to stand up, hunchback, inflexible tail / curled upwards

Body Weight (BW)	
< 90 % of Day 0	
< 85 % of Day 0	
< 80 % of Day 0	Humane endpoint was also reached if the weight of mice reduced more than 5 % on two following days.

			Day after Challenge																					
	Mouse	Parameter	0	1	2	3	4	5	6	7	8	9	10	11	12	13	14	15	16	17	18	19	20	21
Anti-rabies (40 µg mRNA-LNP)	#1	Score	0	0	0	0	0	0	0	0	0	0	0	0	0	0	0	0	0	0	0	0	0	0
		BW	100	96	96	95	95	95	95	95	95	97	94	95	96	97	97	100	100	99	97	96	97	98
	#2	Score	0	0	0	0	0	0	0	0	0	0	0	0	0	0	0	0	0	0	0	0	0	0
		BW	100	95	94	99	97	96	94	95	95	98	99	100	100	103	105	109	110	107	103	102	104	107
	#3	Score	0	0	0	0	0	0	0	0	0	0	0	0	0	0	0	0	0	0	0	0	0	0
		BW	100	96	97	101	102	99	99	98	97	100	98	98	100	98	98	100	102	104	103	105	106	105
	#4	Score	0	0	0	0	0	0	0	0	0	0	1	1	1	1	1	1	0	0	0	0	0	0
		BW	100	96	101	104	104	97	96	96	95	99	96	96	99	99	102	100	102	103	102	104	106	106
	#5	Score	0	0	0	0	0	0	0	0	0	0	0	0	0	0	0	0	0	0	0	0	0	0
		BW	100	88	89	94	97	99	99	99	98	103	104	101	104	106	109	110	109	111	107	105	106	99
	#6	Score	0	0	0	0	0	0	0	1	1	1	1	1	1	1	1	2	2	1	1	1	1	1
		BW	100	98	101	102	95	93	90	90	89	87	88	89	90	95	95	93	95	97	95	96	100	98
	#7	Score	0	0	0	0	0	0	0	0	0	0	0	0	0	0	0	0	0	0	0	0	0	0
		BW	100	93	96	97	93	95	88	89	90	91	92	89	89	91	93	93	96	96	95	99	100	97
	#8	Score	0	0	0	0	0	0	0	0	0	0	0	0	0	0	0	0	0	0	0	0	0	0
		BW	100	96	98	100	101	102	101	103	102	101	102	102	103	105	107	104	106	106	108	112	112	109
Anti-influenza B (40 µg mRNA-LNP)	#1	Score	0	0	0	0	0	0	**3**	N/A	N/A	N/A	N/A	N/A	N/A	N/A	N/A	N/A	N/A	N/A	N/A	N/A	N/A	N/A
		BW	100	93	91	97	99	88	**82**															
	#2	Score	0	0	0	0	0	0	0	0	0	0	0	0	0	0	0	0	0	0	0	0	0	0
		BW	100	98	97	98	98	96	98	99	98	98	96	97	98	96	95	93	92	98	99	99	100	99
	#3	Score	0	0	0	0	0	0	2	**3**	N/A	N/A	N/A	N/A	N/A	N/A	N/A	N/A	N/A	N/A	N/A	N/A	N/A	N/A
		BW	100	95	98	102	102	93	89	**82**														
	#4	Score	0	0	0	0	0	0	1	1	**2**	N/A	N/A	N/A	N/A	N/A	N/A	N/A	N/A	N/A	N/A	N/A	N/A	N/A
		BW	100	92	92	96	96	96	89	80	**72**													
	#5	Score	0	0	0	0	0	0	2	**3**	N/A	N/A	N/A	N/A	N/A	N/A	N/A	N/A	N/A	N/A	N/A	N/A	N/A	N/A
		BW	100	96	97	97	98	92	86	**80**														
	#6	Score	0	0	0	0	0	1	2	N/A	N/A	N/A	N/A	N/A	N/A	N/A	N/A	N/A	N/A	N/A	N/A	N/A	N/A	N/A
		BW	100	93	93	92	95	87	81															
	#7	Score	0	0	0	0	0	0	2	**3**	N/A	N/A	N/A	N/A	N/A	N/A	N/A	N/A	N/A	N/A	N/A	N/A	N/A	N/A
		BW	100	93	93	94	94	88	81	**76**														
	#8	Score	0	0	0	0	0	0	2	**3**	N/A	N/A	N/A	N/A	N/A	N/A	N/A	N/A	N/A	N/A	N/A	N/A	N/A	N/A
		BW	100	94	91	92	91	82	78	**74**														

Appendix Table S4 continued

Supporting information Fig. 4e

Scores	
0	no clinical signs
1	early sign of rabies encephalitis: ruffled fur, excessive grooming, restless / overly active, coordination problems (unsteady gait), blinking/squeezing of eyes, arched back
2	progression to rabies encephalitis: ruffled fur, impaired use of one of the hind legs, inflexible tail, hunchback
3	rabies encephalitis **(humane endpoint)**: impaired use of the hind quarter, rolls over when walking, inability to stand up, hunchback, inflexible tail / curled upwards

Body Weight (BW)	
< 90 % of Day 0	
< 85 % of Day 0	
< 80 % of Day 0	Humane endpoint was also reached if the weight of mice reduced more than 5 % on two following days.

			Day after Challenge																					
	**Mouse**	**Parameter**	**0**	**1**	**2**	**3**	**4**	**5**	**6**	**7**	**8**	**9**	**10**	**11**	**12**	**13**	**14**	**15**	**16**	**17**	**18**	**19**	**20**	**21**
**Anti-rabies (10 µg LNP-mRNA)**	#1	Score	0	0	0	0	0	0	0	0	0	0	0	0	0	0	0	0	0	0	0	0	0	0
		BW	100	97	99	100	100	98	100	100	99	100	99	103	102	101	103	105	103	105	107	106	109	107
	#2	Score	0	0	0	0	0	0	0	0	0	0	0	0	0	0	0	0	0	0	0	0	0	0
		BW	94	94	95	95	95	94	94	97	96	97	97	100	100	100	99	97	96	97	98	97	99	100
	#3	Score	0	0	0	0	0	0	2	3	N/A	N/A	N/A	N/A	N/A	N/A	N/A	N/A	N/A	N/A	N/A	N/A	N/A	N/A
		BW	101	100	102	95	94	86	83	76														
	#4	Score	0	0	0	0	0	0	0	0	0	0	0	0	0	0	0	0	0	0	0	0	0	0
		BW	96	93	100	100	99	93	95	98	97	95	93	95	96	94	97	99	99	100	99	100	97	95
	#5	Score	0	0	0	0	0	1	3	3	N/A	N/A	N/A	N/A	N/A	N/A	N/A	N/A	N/A	N/A	N/A	N/A	N/A	N/A
		BW	101	101	100	99	96	91	85	76														
	#6	Score	0	0	0	0	0	0	0	0	0	0	0	0	0	0	0	0	0	0	0	0	0	0
		BW	100	97	100	101	102	101	102	103	102	102	101	100	102	103	102	104	104	104	102	104	104	103
	#7	Score	0	0	0	0	0	2	3	N/A	N/A	N/A	N/A	N/A	N/A	N/A	N/A	N/A	N/A	N/A	N/A	N/A	N/A	N/A
		BW	99	96	96	99	98	91	84															
	#8	Score	0	0	0	0	0	0	0	0	0	0	0	0	0	0	0	0	1	1	0	0	0	0
		BW	101	96	99	100	103	102	103	103	104	103	103	101	103	104	104	105	105	105	104	105	104	104
**Anti-influenza B (10 µg LNP-mRNA)**	#1	Score	0	0	0	0	0	1	3	N/A	N/A	N/A	N/A	N/A	N/A	N/A	N/A	N/A	N/A	N/A	N/A	N/A	N/A	N/A
		BW	100	100	100	103	103	98	93															
	#2	Score	0	0	0	0	0	1	3	N/A	N/A	N/A	N/A	N/A	N/A	N/A	N/A	N/A	N/A	N/A	N/A	N/A	N/A	N/A
		BW	100	96	94	96	94	85	78															
	#3	Score	0	0	0	0	0	0	0	0	0	0	0	0	0	0	1	0	0	0	0	1	0	1
		BW	100	95	97	99	98	95	95	95	94	94	95	87	88	90	86	85	86	90	94	95	95	97
	#4	Score	0	0	0	0	0	1	3	N/A	N/A	N/A	N/A	N/A	N/A	N/A	N/A	N/A	N/A	N/A	N/A	N/A	N/A	N/A
		BW	100	98	101	104	103	89	84															
	#5	Score	0	0	0	0	0	1	2	3	N/A	N/A	N/A	N/A	N/A	N/A	N/A	N/A	N/A	N/A	N/A	N/A	N/A	N/A
		BW	100	96	99	98	97	86	81	73														
	#6	Score	0	0	0	0	0	0	0	0	0	0	0	0	0	0	0	0	0	0	0	0	0	0
		BW	100	97	100	102	101	100	99	95	96	97	96	95	97	95	99	101	101	100	101	100	101	102
	#7	Score	0	0	0	0	0	0	2	3	N/A	N/A	N/A	N/A	N/A	N/A	N/A	N/A	N/A	N/A	N/A	N/A	N/A	N/A
		BW	100	100	103	103	102	95	88	80														
	#8	Score	0	0	0	0	0	0	0	0	0	0	0	0	0	0	1	0	0	0	0	0	0	0
		BW	100	101	101	99	96	97	99	97	97	94	92	92	96	95	98	100	103	103	102	102	103	106
**PBS**	#1	Score	0	0	0	0	0	1	3	N/A	N/A	N/A	N/A	N/A	N/A	N/A	N/A	N/A	N/A	N/A	N/A	N/A	N/A	N/A
		BW	100	98	96	96	94	83	78															
	#2	Score	0	0	0	0	0	2	3	N/A	N/A	N/A	N/A	N/A	N/A	N/A	N/A	N/A	N/A	N/A	N/A	N/A	N/A	N/A
		BW	100	102	101	99	100	87	80															
	#3	Score	0	0	0	0	0	0	1	3	N/A	N/A	N/A	N/A	N/A	N/A	N/A	N/A	N/A	N/A	N/A	N/A	N/A	N/A
		BW	100	97	96	97	98	90	85	79														
	#4	Score	0	0	0	0	0	3	N/A	N/A	N/A	N/A	N/A	N/A	N/A	N/A	N/A	N/A	N/A	N/A	N/A	N/A	N/A	N/A
		BW	100	101	102	103	93	84																
	#5	Score	0	0	0	0	0	1	3	N/A	N/A	N/A	N/A	N/A	N/A	N/A	N/A	N/A	N/A	N/A	N/A	N/A	N/A	N/A
		BW	100	100	101	103	100	92	83															
	#6	Score	0	0	0	0	0	1	3	N/A	N/A	N/A	N/A	N/A	N/A	N/A	N/A	N/A	N/A	N/A	N/A	N/A	N/A	N/A
		BW	100	97	96	98	98	87	79															
	#7	Score	0	0	0	0	0	1	2	2	N/A	N/A	N/A	N/A	N/A	N/A	N/A	N/A	N/A	N/A	N/A	N/A	N/A	N/A
		BW	100	101	101	99	100	90	83	76														
	#8	Score	0	0	0	0	0	3	N/A	N/A	N/A	N/A	N/A	N/A	N/A	N/A	N/A	N/A	N/A	N/A	N/A	N/A	N/A	N/A
		BW	100	93	92	92	87	79																
