# Supplementary material for: mRNA mediates passive vaccination against infectious agents, toxins, and tumors
Source: EMBO Mol Med. 2017 Aug 9;9(10):1434–47. doi: 10.15252/emmm.201707678 (PMC5623855; doi:10.15252/emmm.201707678)
Supplement: Supplementary file 2 — Source Data for Appendix [file EMMM-9-1434-s003.zip › EMM_07678_SD_Appendix/EMM_07678_SD_FigS1.pdf]

Appendix Figure S1A

Protein standard used: Chameleon DUO, Li-COR (125 kDa indicated where possible)

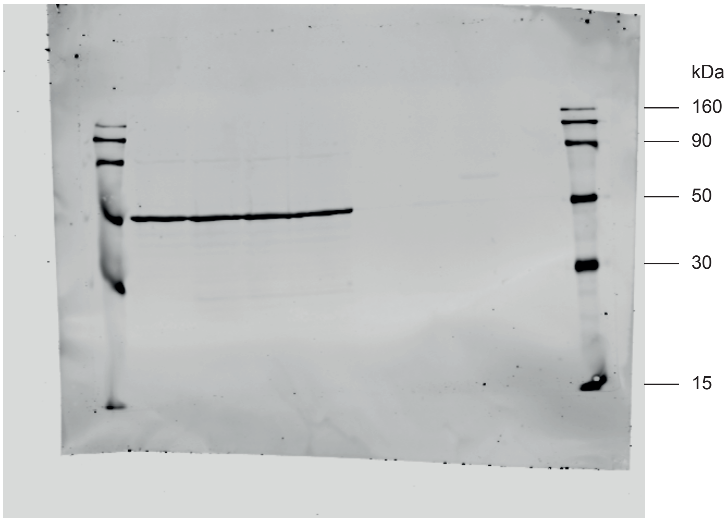

Channel 700
(anti-tubulin)

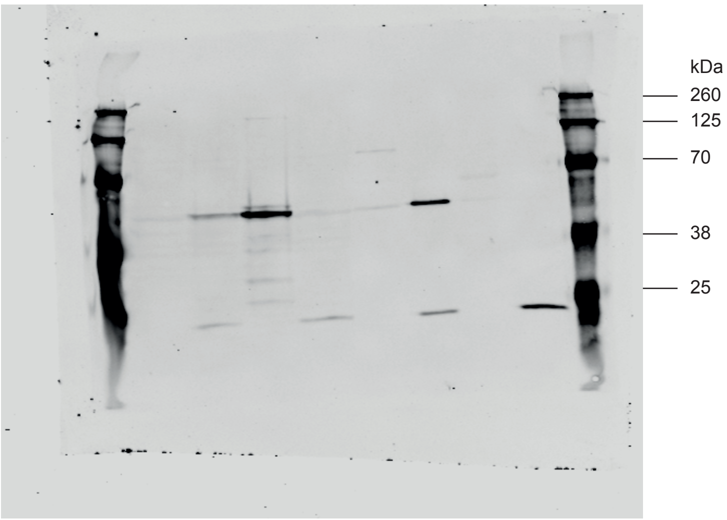

Channel 800
(anti-human IgG H+L)

Appendix Figure S1C

Protein standard used: Chameleon DUO, Li-COR (125 kDa indicated where possible)

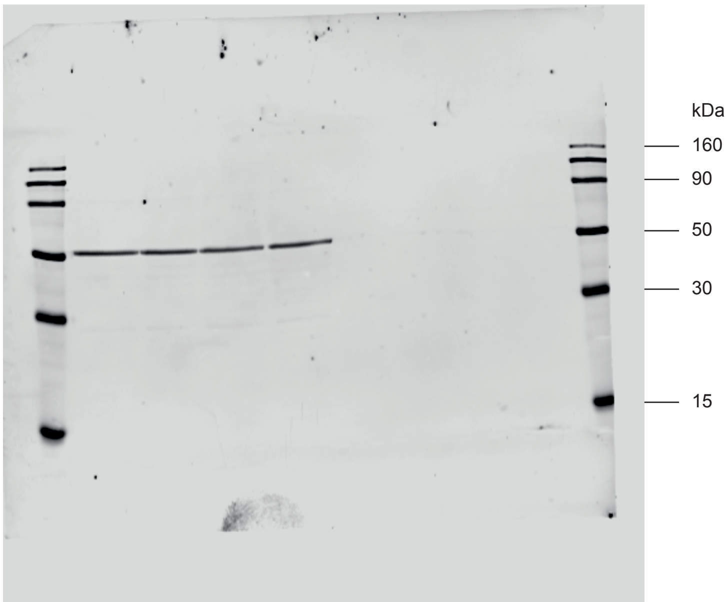

Channel 700
(anti-tubulin)

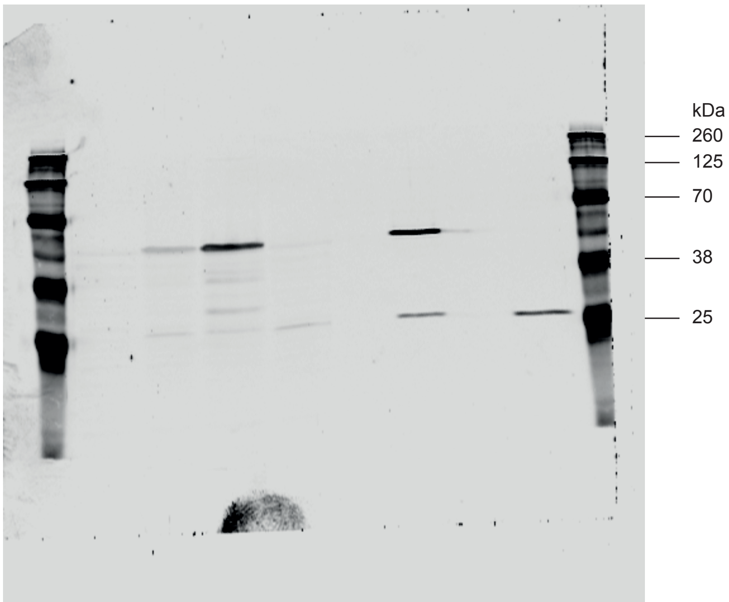

Channel 800
(anti-human IgG H+L)

Appendix Figure S1E

Protein standard used: Chameleon DUO, Li-COR (125 kDa not indicated)

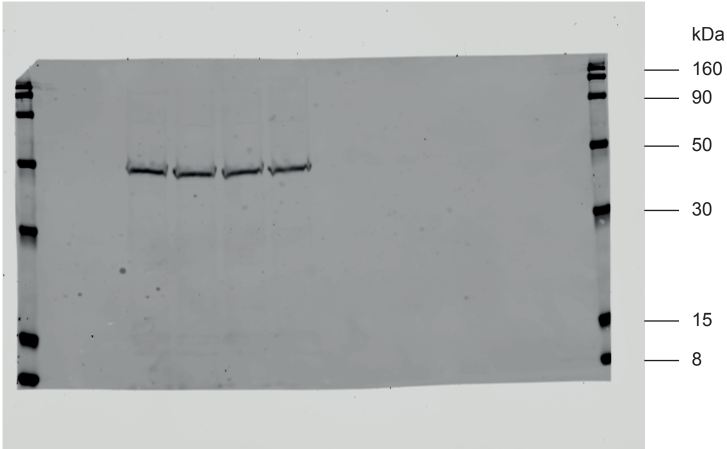

Channel 700
(anti-tubulin)

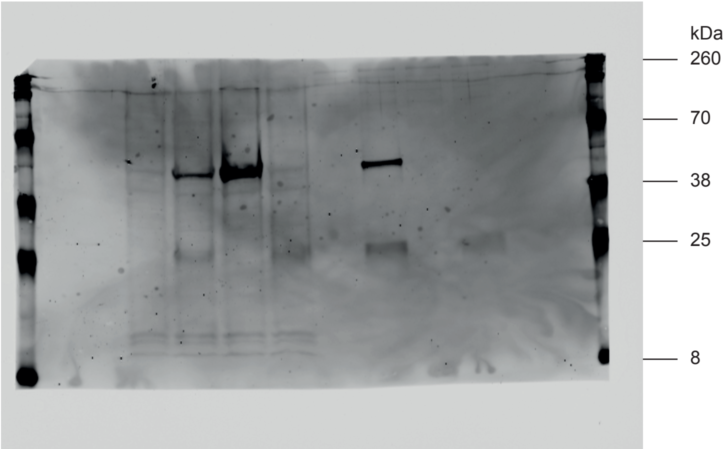

Channel 800
(anti-human IgG H+L)
