# Supplementary figures and images for: mRNA mediates passive vaccination against infectious agents, toxins, and tumors
Source: EMBO Mol Med. 2017 Aug 9;9(10):1434–47. doi: 10.15252/emmm.201707678 (PMC5623855; doi:10.15252/emmm.201707678)

**Appendix Figure S9 (A, B)**

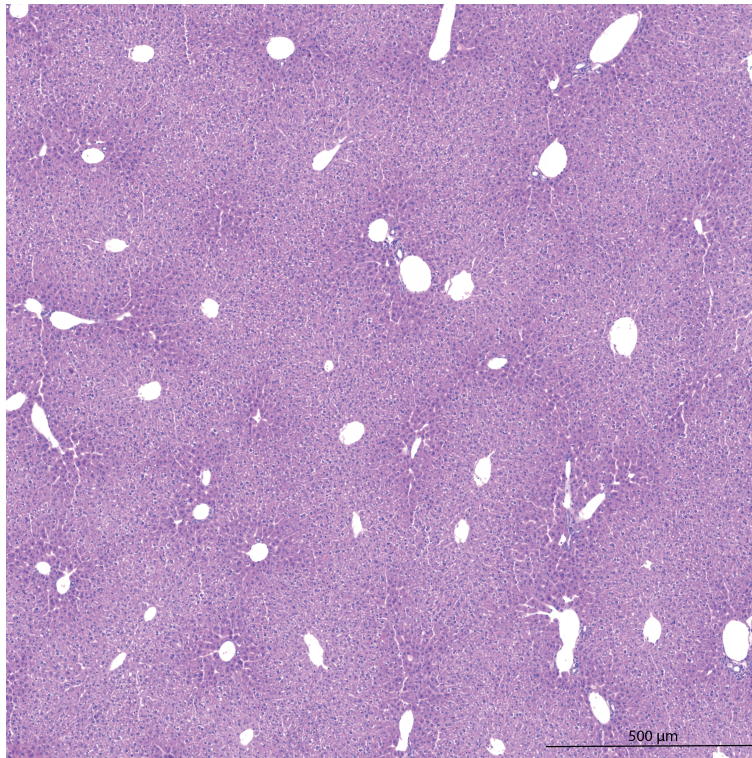

**Appendix Figure S9 (C, D)**

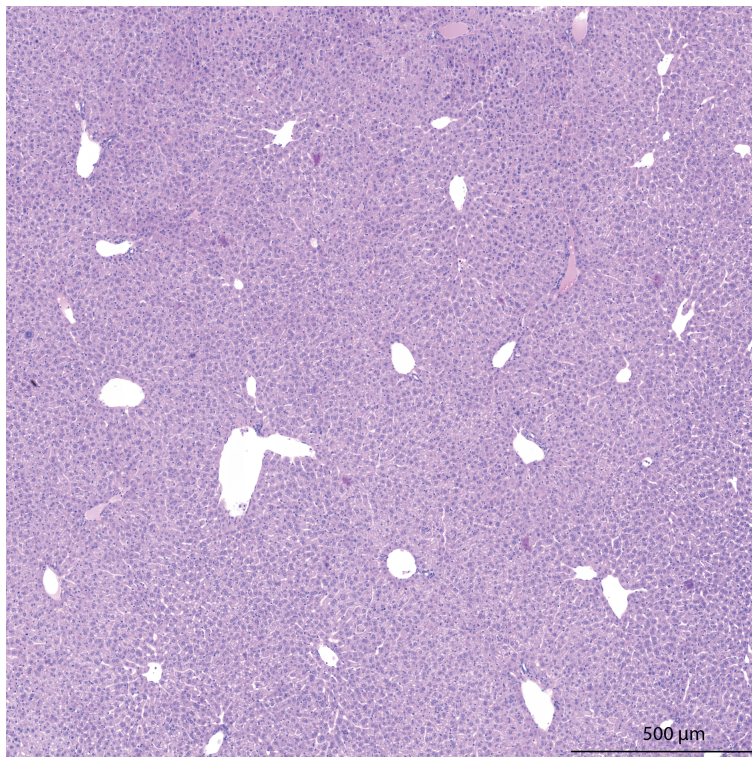

Supplement: Supplementary file 2 — Source Data for Appendix [file EMMM-9-1434-s003.zip › EMM_07678_SD_Appendix/EMM_07678_SD_FigS9.pdf]
