# Supplementary material for: mRNA mediates passive vaccination against infectious agents, toxins, and tumors
Source: EMBO Mol Med. 2017 Aug 9;9(10):1434–47. doi: 10.15252/emmm.201707678 (PMC5623855; doi:10.15252/emmm.201707678)
Supplement: Supplementary file 4 — Source Data for Figure 1 [file EMMM-9-1434-s002.pdf]

Figure 1B

Protein Standard used: Chameleon DUO, Li-COR (125 kDa not indicated)

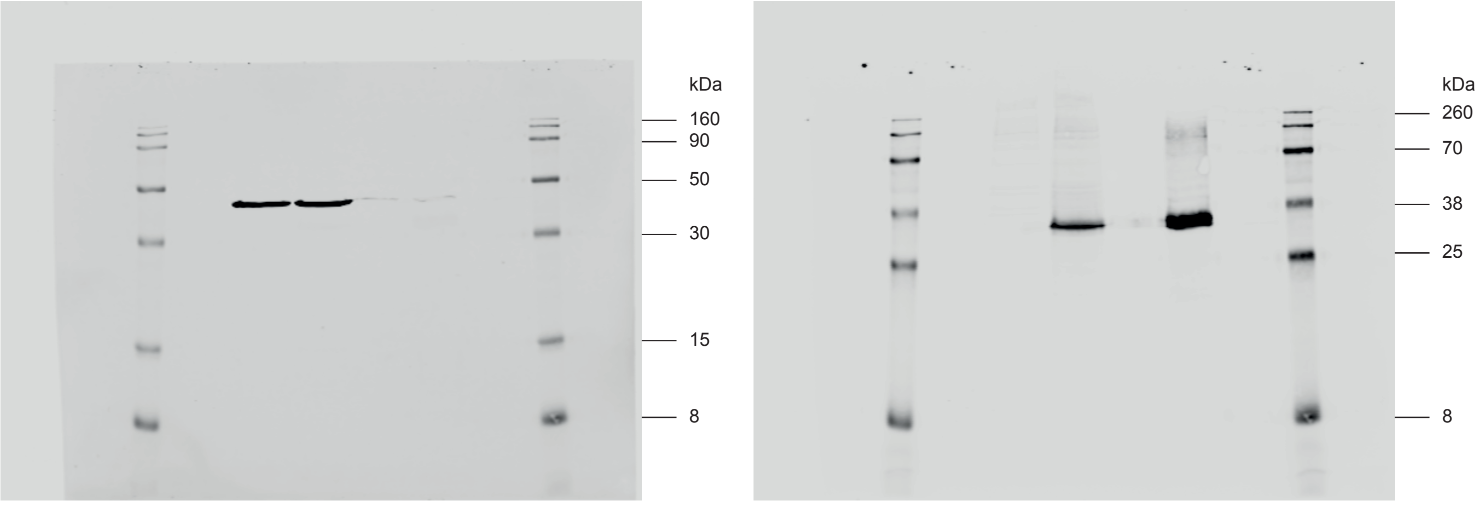

Channel 700
(anti-β-actin)

Channel 800
(anti-E-Tag)

Figure 1E

Protein Standard used: Chameleon DUO, Li-COR

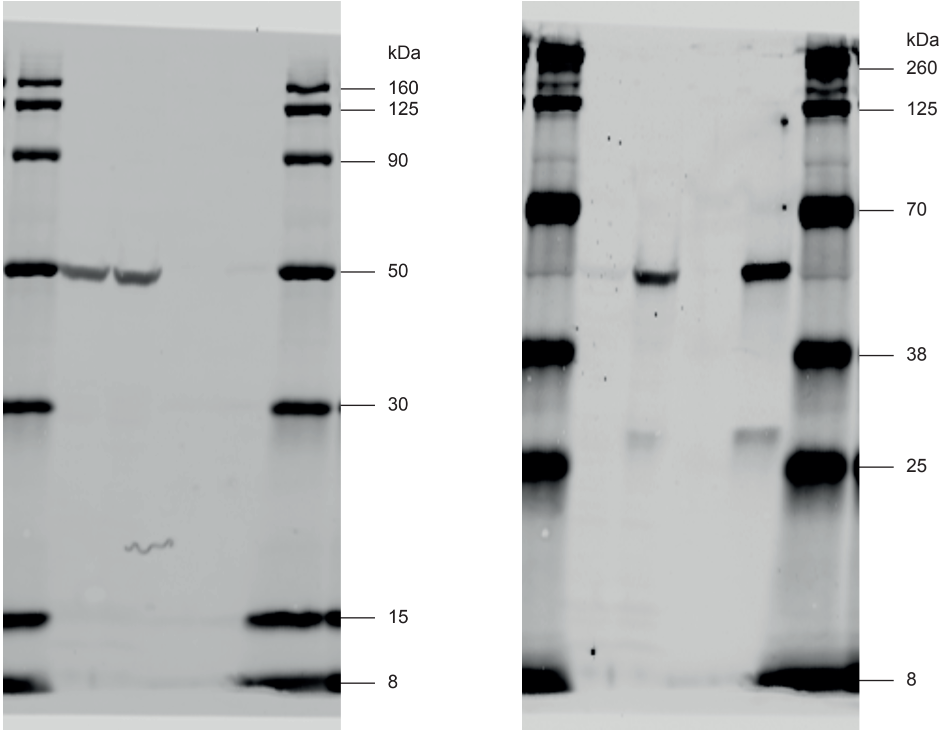

Channel 700
(anti-tubulin)

Channel 800
(anti-human IgG H+L)
